# The Yellow Fever in New Orleans

**Published:** 1854-05

**Authors:** 


*The Yellow Fever in New Orleans.*—We have received from Dr. E. D. Fenner, his history of the late epidemic.

That such a history should be written, that the causes of so fearful a visitation should be thoroughly investigated, is evident, when we look at the records of interments. We have already published these, but the grand total is so enormous that we wish briefly to review it.

The first case occurred on May 23d. Up to June 25th, the whole number of deaths was but 22. From that, for three months ending Sept. 24, the number was 7,664; to which should be added 149 occurring after that time, making a total of 8,813. These are the deaths from yellow fever. The whole number of interments from May 21 to Oct. 8, was 11,100.

The manner in which Dr. Fenner has accomplished his task, indicates much labor, and careful thought. We are pleased to find an accurate meteorological record kept by Dr. Barton. A letter recently received by us from Mr. Lorin Blodgett, who is at the head of the meteorological department of the Smithsonian Institute, has the following relative to this subject:

" Since the publication of the article alluded to,* much corroborative matter has been developed. Dr. Barton, of New Orleans, in investigating the yellow fever district, has fully confirmed its reference to maximum conditions of heat and humidity. In all parts of this district, including the West India Islands, the same atmospheric condition developed the same sanitary results. His report of these comparisons will soon appear."

Dr. Fenner's history enables us to present an abstract of these conditions. We give only such a summary as may subserve our present purpose. In the statement of the dew point, 100 represents the point of saturation; and of course all lesser figures indicate a proportionate decrease of humidity.

| Month. | Av. Dew Point. | Therm'r. Av. Heat. | In. of Rain. | Yellow Fever. Deaths from. |
|---|---|---|---|---|
| January, | 44.93 | 47.20 | 3.190 | 0 |
| February, | 50.48 | 56.05 | 4.600 | 0 |
| March, | 56.17 | 62.43 | 6.870 | 0 |
| April, | 66.60 | 70.39 | 1.848 | 0 |
| May, (great heat for 12 days,) | 67.11 | 73.32 | 3.840 | 1 |
| June, | 73.20 | 80.73 | 1.757 | 46 |
| July, | 72.13 | 79.88 | 11.708 | 1247 |
| August, | 78.08 | 81. | 6.34 | 5625 |
| September, | 70.93 | 76.23 | 5.70 | 852 |

No one can fail to notice here the condition of humidity as compared with the number of deaths. That such a condition should not have drawn Dr. Fenner's attention, excites our wonder. We have been obliged to make up the above table from different parts of this pamphlet. The reader will get but an indifferent idea of the heat by the record of the thermometer. Thus, in June, the greatest heat was 91°, the lowest 70°; in July 89°, and 71°;

* Republished in the Dec. No. of this Journal from the N. Y. Jour. of Med.

in August 72° maximum, and 19° minimum. But it will be seen that the two conditions of heat and humidity coexisted in a high ratio. As the dew point gradually rose above the average point of 50, and continued till it reached so near the point of saturation, as 78 in August accompanied by an average temperature of 81°, the mortality went on to its highest figure of 5624 in one month. It is expected that the present year will afford us full records of these conditions for the whole country. Comparisons can then be instituted and we may then reach the truth. H.

